# Fetal behavior and gestational serotonin reuptake inhibitor exposure: relationships between behavior, drug dosage, plasma drug level, and a measure of drug bioeffect

**DOI:** 10.1038/s41386-024-01923-1

**Published:** 2024-08-10

**Authors:** Amy L. Salisbury, George M. Anderson, Amy Yang, Catherine S. Stika, Laura J. Rasmussen-Torvik, Jacqueline K. Gollan, Katherine L. Wisner

**Affiliations:** 1 School of Nursing, Virginia Commonwealth University, Richmond, VA 23298-0567 USA; 2 Department of Pediatrics, Women & Infants Hospital, Alpert Medical School at Brown University, Providence, RI USA; 3 Child Study Center and the Department of Laboratory Medicine, Yale University School of Medicine, 230 S. Frontage Rd., New Haven, CT 06525 USA; 4 AY Analytics, Chicago, IL 60611 USA; 5 Department of Obstetrics and Gynecology, Northwestern University Feinberg School of Medicine, 676 North St Clair Street, Suite 1000, Chicago, IL 60611 USA; 6 Department of Preventive Medicine, Northwestern University Feinberg School of Medicine, 680 N Lake Shore Drive, Suite 1400, Chicago, IL 60611 USA; 7 Department of Psychiatry and Behavioral Sciences, Northwestern University Feinberg School of Medicine, 676 North St Clair Street, Suite 1000, Chicago, IL 60611 USA; 8 Children’s National Hospital, Developing Brain Institute, 111 Michigan Ave., NW, 20001 Washington, DC USA

**Keywords:** Predictive markers, Outcomes research

## Abstract

Determination of the relationships between drug dosage, maternal and infant (cord blood) plasma drug concentrations, and serotonin reuptake inhibitor (SRI) bioeffect on offspring neurobehavior is crucial to assessing the effects of gestational SRI exposure. Measurement of maternal and cord blood platelet serotonin (5-HT) provides an index of inhibitory bioeffect at the 5-HT transporter and complements other measures of drug exposure. Three groups of mother-infant pairs were evaluated: (1) mothers with depression untreated with SRIs (DEP, *n* = 17), (2) mothers treated for depression with SRIs (DEP + SRI, *n* = 17), and (3) mothers who were not depressed and untreated (ND, *n* = 29). Fetal movement was assessed using a standardized ultrasound imaging and rating protocol. Maternal and cord blood platelet 5-HT levels were obtained from all participants. For the SRI + DEP group, maternal and infant plasma drug concentrations and an estimate of third-trimester maternal SRI drug exposure were obtained. As expected, substantially lower median platelet 5-HT levels were observed in the DEP + SRI group than in the non-exposed, combined ND and DEP groups. In non-exposed mothers and infants, platelet 5-HT levels were not affected by the presence of maternal depression. Lower maternal and infant platelet 5-HT levels were associated with more immature fetal movement quality. Although these data are limited by small sample size, the bioeffect index of in vivo platelet 5-HT transporter inhibition appears to provide a valuable approach for elucidating and possibly predicting the effects of gestational SRI exposure on fetal and perinatal neurobehavior.

## Background

In the U.S., Major Depressive Disorder (MDD) occurs in ~7.5% of individuals during pregnancy and ~6.5% of individuals during early postpartum [1]. Without treatment, they have an elevated risk for poor health and adverse fetal and newborn outcomes, including preterm birth and reduced gestational size [2, 3] These findings underscore the importance of treating perinatal depression. Serotonin reuptake inhibitor (SRI) antidepressants [including selective serotonin reuptake inhibitors (SSRIs) and serotonin-norepinephrine reuptake inhibitors (SNRIs)] are the first-line medications for pregnant persons diagnosed with depression and are frequently prescribed [4, 5].

However, near-term SRI exposure confers a risk ratio of 3.0 (95% CI, 2.0–4.4) for Neonatal Adaptation Syndrome (NAS) vs. early or no pregnancy exposure. Exposed neonates often display neurobehavioral, respiratory, and GI signs. In addition the rate of NICU admission is significantly higher in SRI-exposed vs. unexposed neonates at 13.7% vs. 8.2%, respectively [OR = 1.5 (95% CI, 1.4–1.5)] [6]. Thus, balancing maternal mental health benefits with risks to fetal and infant health during treatment with SRIs is a clinical challenge. Characterization of the effects of SRI exposure on fetal and infant neurobiology, physiology and behavior is needed to improve the care of childbearing individuals.

Fetal exposure to SRIs has the potential to alter serotonin signaling in the developing central nervous and other organ systems. A large body of research has examined the effects of prenatal SRI on the developing brain using neuroimaging and through the use of animal studies [7–10]. Our group and others have reported the effects of SRI exposure on fetal and infant behavior [9, 11–13]. In the present study, we examined the relationships between fetal behavior and measures of SRI exposure including estimated third-trimester SRI use, maternal and newborn plasma drug concentrations, and maternal and newborn platelet serotonin (5-HT) levels (expressed both as ng/mL and ng per billion platelets).

The platelet 5-HT measure reflects the extent of inhibition of the 5-HT transporter (SERT) and has been used in many studies to assess SRI bioeffect [14–16]. Platelets absorb 5-HT via the membrane SERT throughout their 8-10-day lifespan [17, 18] and blockade or inhibition of SERT results in lower levels of platelet 5-HT [14]. Based on theoretical considerations, platelet 5-HT should provide a better index of SRI bioeffect than total plasma drug concentrations or estimations of drug use. First, platelet 5-HT is affected by the free, physiologically active concentration of the SRI in plasma, and this free pool is predominant in determining brain levels of the drug [19, 20]. Second, inhibitory effects at the platelet SERT reflect the cumulative effect not only of the parent drug but also of all active metabolites. Third, the platelet 5-HT measure reflects the SRI inhibitory bioeffect at SERT over the platelet’s lifespan of 8 to 10 days. Thus, platelet 5-HT provides a time-averaged index of SERT inhibition that is less affected by variation in protein binding, the timing of drug administration, recent adherence to prescribed dosage, and variations in blood draw timing. The validity and utility of the platelet 5-HT measure is empirically supported by neuroimaging studies that find SRI occupancies of brain SERT of 75-90% with clinical doses of SRIs, findings very similar to the reductions seen in platelet 5-HT after administration of SRIs [10].

We tested four main hypotheses: (1) Platelet 5-HT concentrations observed for SRI-exposed mothers and infants will be substantially lower than in non-exposed individuals. (2) Platelet 5-HT concentrations from non-exposed depressed mothers or their infants will not differ from those of non-exposed non-depressed individuals. (3) Maternal and infant platelet 5-HT will be negatively associated with SRI concentration levels at birth, and (4) Lower maternal and neonatal platelet 5-HT levels (greater SRI bioeffect) will be associated with measures of lower fetal quality of movement (shorter quiescent bout lengths and more spontaneous fetal activity). We additionally tested if significant  associations between the platelet bioeffect and behavioral measures were greater than associations between the plasma drug levels or drug exposure estimates and the behavioral measures. Finally, we also compared 5-HT levels in the maternal and infant groups, and examined the observed heritability of platelet 5-HT in the non-drug exposed mother-infant pairs.

### Participants and methods

#### Participants/recruitment

Pregnant women were recruited from community obstetric practices to participate in a prospective longitudinal naturalistic cohort study. Eligibility criteria included age 18–40 years, English or Spanish speaking, 23- to 34-week singleton gestation, negative urine drug screen, and average alcohol consumption of less than one-half drink/day. We also required smoking less than five cigarettes/day in the first trimester and no smoking in the second or third trimesters (confirmed with urine cotinine screen). All participants had a standard 2nd trimester medical ultrasound before participating to rule out fetal medical conditions or anomalies that would confound study findings or result in frequent ultrasound imaging for monitoring.

Participants attended an intake interview and up to two fetal assessments between 26–30 weeks and 32–36 weeks gestation. Diagnostic interviews and self-report assessments were conducted. At delivery, maternal venous and umbilical cord blood were collected to determine plasma SRI and platelet 5-HT concentrations. Participants were compensated for their time ($150). All mothers provided written informed consent. The Women & Infants’ Hospital Institutional Review Board reviewed and approved the study.

**Fig. 1:**
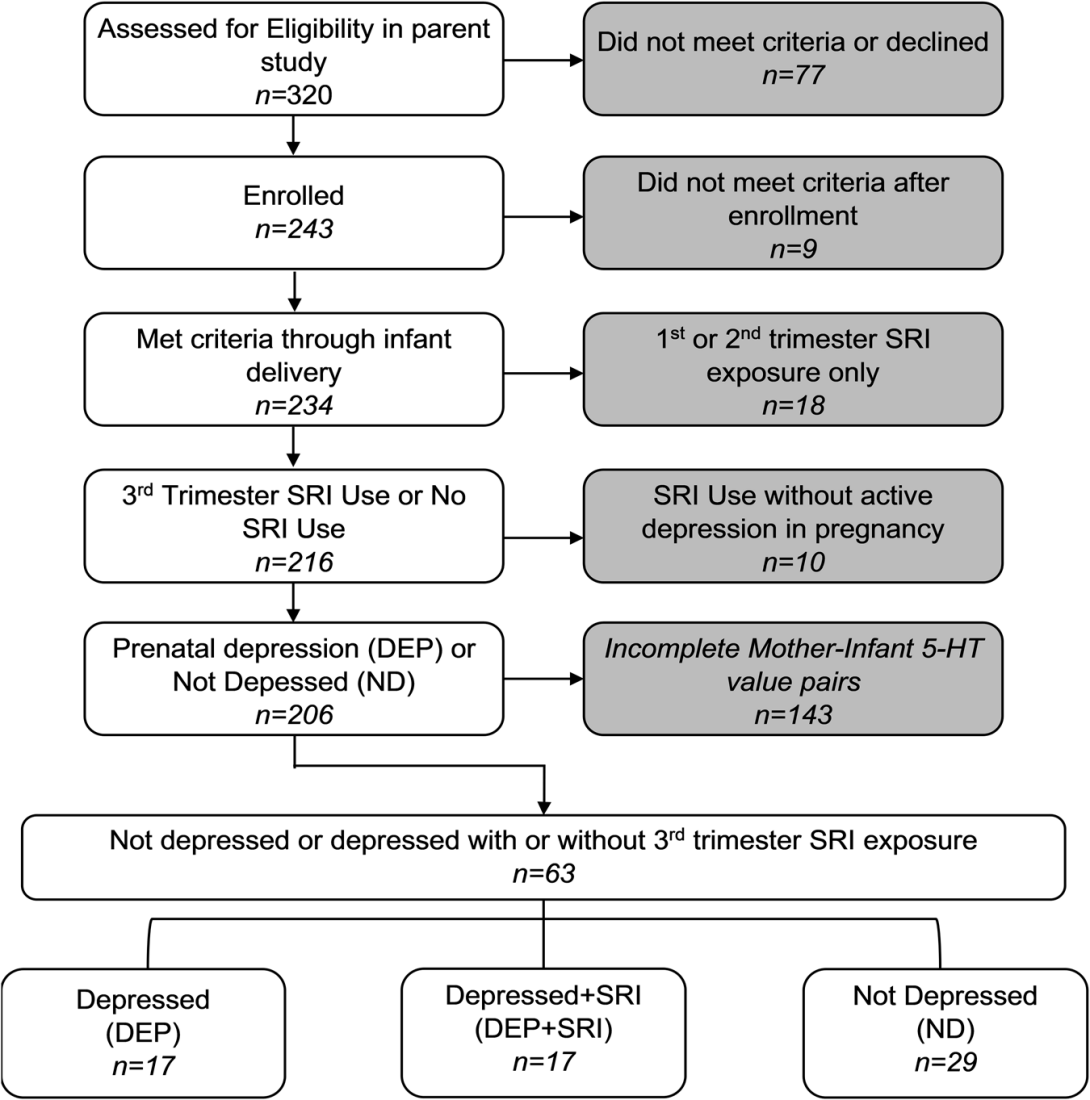
Flow diagram for subject selection and participation. DEP depression only, DEP + SRI mothers with depression and SRI treatment, ND mothers without depression or SRI treatment, 5-HT serotonin.

The parent study has been described elsewhere [13]. Briefly, mothers were self-referred from community obstetric care provider offices and were screened by telephone. As depicted in Fig. 1, for inclusion in this analysis, all participants in the depression groups were required to have a diagnosis of depression with or without third-trimester SRI treatment and with platelet 5-HT concentrations determined for both the mother and infant. These criteria resulted in a sample of 63 individuals. Individuals were assigned to one of three groups based on their depression and SRI-exposure status: (1) depressed mothers untreated with medication (DEP group, *n* = 17), (2) mothers treated for depression with SRI (DEP + SRI group, *n* = 17), and (3) untreated non-depressed mothers (ND group, *n* = 29).

#### Descriptive measures

Demographic data included the Hollingshead index of socioeconomic status (SES), with categories 4 and 5 combined to indicate low SES. Maternal psychiatric diagnoses, depression severity, and treatment were assessed at each prenatal visit and 30 days post delivery. Measures included the Structured Clinical Interview for DSM-IV-I. All episodes of depression throughout the pregnancy and the first month postpartum were identified with onset and offset dates to determine the total duration of depressive episodes. The Inventory of Depressive Symptomatology – Clinician Rated (IDS-C), a 30-item semi-structured interview, was used to assess the severity of symptoms [21]. The IDS-C has established high internal and concurrent validity and sensitivity, with the following continuous total score and severity levels; None: 0–11, Mild: 12–23, Moderate: 24–36, Severe: 37–84 [22]. Anxiety was measured using the Hamilton Anxiety Rating Scale (HAM-A), score range = 0–56; Mild <17, Moderate: 18–24, severe: 25–30 [23]. A calendar-based interview assessment based on the Timeline Follow Back Method was used to measure amount and adherence with prescribed medications [24, 25]. Maternal reports of all psychotropic medications (SRIs, other antidepressants, benzodiazepines, antipsychotics, mood stabilizers) including dosage and the number of days taken for each week of the pregnancy, were obtained. Participants provided urine for testing for substance exposure with a 10-drug panel and a nicotine/cotinine strip (*Craig Medical)*.

#### Classification of subgroups

Participants in the ND group did not meet diagnostic criteria for any psychiatric disorder at any time in pregnancy, and scored less than 24 (none to mild symptoms) on the IDS-C at both administrations and reported no use of psychotropic medications during pregnancy. Women were categorized as DEP if they were diagnosed with a unipolar major depressive disorder during the 2nd and/or 3rd trimester of this pregnancy or if they scored 24 or greater on the IDS-C across both pregnancy time points and did not use any psychotropic medication during the pregnancy. The DEP + SRI group was defined as women meeting the DEP criteria and reported SRI antidepressant use for at least three consecutive weeks in the third trimester.

#### Plasma drug and whole blood serotonin measures

Maternal and newborn plasma drug concentrations, and maternal and newborn whole blood 5-HT levels (expressed as ng/mL) were measured from maternal venous and infant cord blood in all three groups. 5-HT concentrations were also expressed on a ng/billion platelet basis in order to examine the relationship between the two expressions of 5-HT concentration. The ng/mL value requires only one measurement and is the more widely used expression. While the ng/billion value can potentially account for individual differences in platelet counts, it introduces the variance associated with the measurement of the platelet count and, in this instance, its use reduces the sample number.

A minimum of 3 mL of cord blood was collected immediately after delivery and distributed into sterile lithium heparin tubes and stored at −80 °C until analysis for 5-HT using a published high-performance liquid chromatographic (HPLC) method [16, 26]. A manual whole blood platelet count was obtained for both maternal and infant samples. Maternal and infant plasma was prepared by centrifugation for 10 min at 3000 rpm and stored at −80 °C until analysis of SRI concentrations using a published HPLC method [27]. All analytes were measured with within-assay and assay-to-assay coefficients of variation of less than 7%. Sertraline-equivalent plasma concentrations were calculated for the three patients receiving fluoxetine, escitalopram or citalopram by multiplying the measured concentration of each drug by the minimum of sertraline’s published therapeutic range divided by the minimum of the drug’s published therapeutic range.

#### Estimated third trimester SRI exposure

A Standard Dose Equivalent (SDE) was calculated by dividing the reported SRI dose (in mg/day) by the standard starting dose for each SRI, resulting in an SDE for each day of drug exposure. The approach is similar to that commonly used to calculate imipramine equivalents [28]. The SDEs was then multiplied by the number of days at that dose and those values summed to give a total estimated third trimester maternal SRI exposure in SDE days. The SDE measure is expected also to provide an index of third trimester fetal SRI exposure. The total mean third trimester SRI exposure in SDE days for the 17 mothers of the DEP + SRI group was 119.1 ± 86.1.

#### Fetal neurobehavioral assessment

The Fetal Neurobehavioral Coding System (FENS), was used to measure specific fetal movement patterns between the hours of 12:00–5:00 pm to account for variability in fetal activity levels across the day [29, 30]. Assessments conducted between 28- and 38-week gestational age (*M* = 31.8 ± 2.8 weeks) were used in the present analyses to evaluate relationships more proximal to biological measures at birth. Participants fasted for 1.5 hours before their appointment and had a standardized snack 30 min prior to the recording. Consistent with guidelines of National Institutes of Child Health and Human Development Workshop Report on Electronic Fetal Monitoring [31], a standard Biophysical Profile (BPP) [32, 33] and Stress test of general fetal health was conducted before the FENS recording [31] The fetus was observed by real-time ultrasound using a Siemens Accuson S2000 ultrasound machine with a 3.75 MHz linear-array transducer. Longitudinal views of the fetal face, trunk, and upper limbs were video recorded on the lowest possible power settings for a maximum of 50 min, which consisted of 4 blocks of a standardized scanning procedure for 7 min interspersed with 3-min rest periods to minimize prolonged and stagnant ultrasound waves.

Video recordings were later coded by trained raters blind to participant study group to document the presence of specific fetal behaviors using the Mangold INTERACT coding program (Mangold International Inc., Atlanta, GA). Full details have been published [29]. Briefly, video recordings were viewed in regular intervals to observe bouts of movement for specific behaviors including head, trunk, and/or limbs movements and their smooth or jerky quality; breathing movements (regular, irregular, vigorous, hiccup); and mouthing movements (non-rhythmic, rhythmic, yawn). The absence of spontaneous movement for one interval was coded as quiescence and this period marked the ending of the previous movement bout. Inter-rater reliability was assessed by comparing scores of at least two coders on 10% of a randomly selected list of fetuses, with intraclass correlations of 0.70–0.97.

#### Statistical analytical plan

Group differences tested for Hypotheses 1 and 2 were tested with ANOVA. Associations tested for Hypotheses 3 and 4 were conducted for exposed mothers and infants. Variable distributions were tested, with fetal behavior and some biomeasure distributions were found to be positively skewed. Therefore, relationships between platelet 5-HT SRI concentrations, and fetal behavior outcomes, were tested with Spearman’s correlation tests, as they are robust to non-linear trends. Hypothesis testing was completed to test specific directions of effect for associations between biological measures and to fetal behavior, therefore a one-tailed significance level of *p* < 0.05 was used to determine statistical significance. Corrections for multiple comparisons were not conducted due to the relatively small sample size. Data analyses were performed using Python programming language 3.10.11 with the assistance of NumPy 1.23.3, SciPy 1.10.1 and Tableone 0.8.0.

## Results

### Descriptive statistics

The descriptive statistics of the total study sample and the three groups (DEP, SRI + DEP, and ND) are given in Table 1. Several demographic and clinical variables significantly differed between groups. These demographic variables included ethnicity, number of pregnancies and deliveries, ancestry, income, and number of living children. Clinical ratings of anxiety and depression also varied across groups. Of note is that none of the participants reported use of alcohol beyond the first trimester. In the SRI + DEP group, it is notable that *N* = 7 (41.2%) of the mothers in the SRI + DEP group reported taking at least one additional psychotropic medication for at least 14 days. The demographic variables were not significantly correlated with or did not differentiate the fetal neurobehavioral outcomes; therefore, they were not included as covariates in the correlation analyses.

**Table 1:** Demographic and clinical characteristics of mothers.

		Group				
		Total	DEP + SRI	DEP	ND	*P* value
		*n* = 63	*n* = 17	*n* = 17	*n* = 29	
		*n* (%) or Mean (±SD)				
Maternal age group	21–24	11 (17.5)	2 (11.8)	6 (35.3)	3 (10.3)	0.36
	25–29	18 (28.6)	5 (29.4)	5 (29.4)	8 (27.6)	
	30–34	15 (23.8)	6 (35.3)	3 (17.6)	6 (20.7)	
	<21	8 (12.7)	1 (5.9)	1 (5.9)	6 (20.7)	
	> = 35	11 (17.5)	3 (17.6)	2 (11.8)	6 (20.7)	
Marital status	Married	35 (55.6)	8 (47.1)	9 (52.9)	18 (62.1)	0.594
	Single	28 (44.4)	9 (52.9)	8 (47.1)	11 (37.9)	
Ethnicity	Hispanic	23 (36.5)	1 (5.9)	11 (64.7)	11 (37.9)	0.002
	Non-Hispanic	40 (63.5)	16 (94.1)	6 (35.3)	18 (62.1)	
Race	Black	7 (11.7)	4 (23.5)	1 (6.7)	2 (7.1)	0.036
	Other	18 (30.0)	1 (5.9)	8 (53.3)	9 (32.1)	
	White	35 (58.3)	12 (70.6)	6 (40.0)	17 (60.7)	
Education	Some schooling	6 (9.5)	3 (17.6)	1 (5.9)	2 (6.9)	0.064
	High school/GED	12 (19.0)	3 (17.6)	6 (35.3)	3 (10.3)	
	Partial college	21 (33.3)	5 (29.4)	8 (47.1)	8 (27.6)	
	College	13 (20.6)	5 (29.4)	1 (5.9)	7 (24.1)	
	Professional	11 (17.5)	1 (5.9)	1 (5.9)	9 (31.0)	
Income	<30k	28 (45.9)	7 (41.2)	9 (52.9)	12 (44.4)	0.01
	30k–50k	9 (14.8)		6 (35.3)	3 (11.1)	
	50k–75k	14 (23.0)	5 (29.4)	2 (11.8)	7 (25.9)	
	75k–99k	3 (4.9)			3 (11.1)	
	> = 100k	7 (11.5)	5 (29.4)		2 (7.4)	
Depression severity (IDS-C)		19.6 (11.1)	29.6 (12.1)	21.2 (7.6)	12.3 (6.1)	<0.001
Anxiety level (HAM-A)	Mild/Mod. Anxi.	18 (29.5)	11 (64.7)	4 (23.5)	3 (11.1)	<0.001
	No Anxiety	39 (63.9)	2 (11.8)	13 (76.5)	24 (88.9)	
	Severe Anxiety	4 (6.6)	4 (23.5)			
Concomitant Psychotropic Meds	No	56 (88.9)	10 (58.8)	17 (100)	29 (100)	<.001
	Yes	7 (11.1)	7 (41.2)	0	0	
Number of pregnancies	1	29 (46.0)	6 (35.3)	5 (29.4)	18 (62.1)	0.005
	2	15 (23.8)	1 (5.9)	7 (41.2)	7 (24.1)	
	>2	19 (30.2)	10 (58.8)	5 (29.4)	4 (13.8)	
Number of deliveries	0	39 (61.9)	8 (47.1)	8 (47.1)	23 (79.3)	0.016
	1	16 (25.4)	4 (23.5)	8 (47.1)	4 (13.8)	
	> = 2	7 (11.1)	5 (29.4)	1 (5.9)	1 (3.4)	
	Unknown	1 (1.6)			1 (3.4)	
Number of living children	0	38 (60.3)	8 (47.1)	7 (41.2)	23 (79.3)	0.006
	1	17 (27.0)	4 (23.5)	9 (52.9)	4 (13.8)	
	> = 2	8 (12.7)	5 (29.4)	1 (5.9)	2 (6.9)	
Delivery mode	Emergent C-sect.	10 (15.9)	2 (11.8)	4 (23.5)	4 (13.8)	0.235
	Planned C-sect.	6 (9.5)	3 (17.6)	3 (17.6)		
	Vaginal	46 (73.0)	12 (70.6)	10 (58.8)	24 (82.8)	
	UNK C-section	1 (1.6)			1 (3.4)	
Apgar 1-min	< = 7	12 (19.4)	6 (35.3)	3 (17.6)	3 (10.7)	0.126
	>7	50 (80.6)	11 (64.7)	14 (82.4)	25 (89.3)	
Apgar 5-min	< = 7	3 (4.8)	2 (11.8)		1 (3.6)	0.255
	>7	59 (95.2)	15 (88.2)	17 (100.0)	27 (96.4)	
Infant gender	Female	32 (50.8)	8 (47.1)	10 (58.8)	14 (48.3)	0.738
	Male	31 (49.2)	9 (52.9)	7 (41.2)	15 (51.7)	

*IDS-C* Inventory of Depressive Symptomatology – Clinician rated, *HAM-A* Hamilton Anxiety Scale.

### Platelet 5-HT levels

The median values IQR for maternal and cord blood platelet 5-HT levels are presented in Table 2. The results of group and subgroup comparisons are described below.

**Table 2:** Group platelet serotonin concentrations: Median (IQR).

	GROUPS				GROUP COMPARISONS	
	ND (*n* = 29)	DEP (*n* = 17)	DEP&ND combined (*n* = 46)	DEP + SRI (*n* = 17)	ND v. DEP *p*-value	DEP&ND v. DEP + SRI *p*-value
	**Mean (Range)**					
Maternal Platelet 5-HT (ng/mL)	133 (107–166)	165 (112–203)	141 (108–180)	17.0 (10.1–27.2)	0.39	<0.001
Maternal Platelet 5-HT (ng/billion)	657 (590–884)	796 (548–942)	719 (576–901)	97.0 (52.2–179)	0.76	<0.001
Infant Platelet 5-HT (ng/mL)	75.0 (52.6–92.9)	83.3 (50.1–113)	78.8 (50.7–97.9)	21.0 (14.7–34.2)	0.50	<0.001
Infant Platelet 5-HT (ng/billion)	391 (265–532)	328 (245–497)	383 (240–527)	109 (65.7–138)	0.40	<0.001

DEP vs. ND Comparisons: 5-HT levels, whether expressed as ng/mL or ng/billion platelets, did not significantly differ between the DEP and ND groups for both maternal and infant samples. Given the observed similarities of the DEP and ND groups, the groups were combined to form a non-SRI exposed (DEP&ND) group.

DEP + SRI vs. combined ND&DEP comparisons: 5HT values in the DEP + SRI group were significantly lower than in the combined non-exposed DEP&ND group (Table 2). This SRI effect on platelet 5-HT was observed for both mothers and infants, and with both units of 5-HT concentration. Comparison of the medians in the DEP&ND and DEP + SRI indicate that SRI exposure resulted in approximately 88% (ng/mL) and 87% (ng/billion) lower maternal platelet 5-HT concentrations. While values in SRI-exposed infants were also lower than in non-exposed infants, the medians were only 73% (ng/mL) and 72% (ng/billion) lower in the exposed infants.

Maternal vs. Infant Comparisons: Within-group comparisons of maternal and infant 5-HT levels in the ND and in the DEP groups demonstrated that infant values were approximately half of their mothers’ values (Table 2) for 5-HT concentrations expressed as ng/mL or ng/billion. In contrast, infant concentrations in the DEP + SRI group were similar to their mothers’ values.

### Plasma drug concentrations

Most mothers in the DEP + SRI group received sertraline. The mean maternal level of sertraline at delivery was 32.1 ± 34.0 ng/mL (*n* = 14). Three mothers received fluoxetine (51.2 ng/mL), escitalopram (16 ng/mL), or citalopram (22.2 ng/mL). These concentrations were converted to sertraline equivalents, for a final combined (sertraline and sertraline equivalent) mean of 31.1 ± 30.7 ng/ml (*n* = 17). Mean plasma sertraline levels in infants of mothers receiving sertraline were 10.9 ± 10.4 ng/mL (*n* = 14). Levels in the infants of mothers taking fluoxetine, escitalopram and citalopram were 12.9 ng/mL, 9.7 ng/mL, and 23.8 ng/mL, respectively. The mean sertraline/sertraline equivalent level in the infants was 11.8 ± 9.8 ng/ml (*n* = 17).

### Inter-correlations of drug exposure/bioeffect measures

In both the mother and infant groups, the ng/mL and ng/billion platelet 5HT measures were found to be highly correlated: Maternal 5-HT ng/mL : maternal 5-HT ng/billion (*r* = 0.96, *p* < 0.001), and infant 5-HT: infant 5-HT ng/billion (*r* = 0.80, *p* < 0.001). Therefore, the simpler and more numerous ng/mL values were used for subsequent analyses of possible behavioral associations. The correlations observed for the various drug exposure and drug bioeffect measures in the SRI + DEP group are presented in Table 3. As predicted, maternal 5-HT ng/mL was negatively correlated with maternal plasma SRI concentration with lower maternal 5-HT levels associated with higher SRI drug levels. Infant 5-HT was also negatively correlated with infant plasma drug concentrations. The estimated third-trimester SRI use in SDE days was negatively correlated with the infant and maternal 5-HT measures, and positively correlated with maternal and infant SRI concentrations.

**Table 3:** Correlation table of platelet serotonin and drug exposure measures.

		INFANT 5-HT ng/mL	MATERNAL PLASMA SRI ng/mL	INFANT PLASMA SRI ng/mL	EST. SRI 3rd TRIM. EXPOSURE
**MATERNAL 5-HT ng/mL**	r_S_ 1-tail p *(n)*	0.826 <0.001 (17)	−0.598 0.006 (17)	−0.398 0.071 (15)	−0.613 0.004 (17)
**INFANT** **5-HT** **ng/mL**	r_S_ 1-tail p *(n)*		−0.656 0.002 (17)	−0.466 0.040 (15)	−0.593 0.006 (17)
**MATERNAL PLASMA SRI** **ng/mL**	r_S_ 1-tail p *(n)*			0.911 <0.001 (15)	0.543 0.012 (17)
**INF** **PLASMA** **SRI** **ng/mL**	r_S_ 1-tail p *(n)*				0.487 0.033 (15)

r_S_ Spearman’s correlation.

To obtain estimates of heritability unaffected by drug effects, non-exposed ND + DEP group correlations of maternal platelet 5-HT with infant platelet 5-HT (ng/mL) were found to be 0.139 (*p* = 0.193).

### Biomeasure-fetal behavior correlations

Fetal gestational age at the behavioral assessment for the exposed group was 34.1 (SD = 1.2; range 31.9–36.1) mean weeks. Fetal behavior measures were not associated with gestational age (Spearman r’s −0.12 to +0.13, 1-tailed *p* values > 0.5) and were not different by fetal sex (*F* values 0–2.5, *p* values 0.13–0.99). The correlation matrix observed for the biomeasures vs. the fetal behavior scores is presented in Table 4. Lower maternal 5-HT concentrations (greater bioeffect) were associated with greater fetal activity (*r* = −0.456, *p* = 0.033) and with shorter periods of quiescence (*r* = −0.431, *p* = 0.042). Similarly, lower infant 5-HT levels were associated with greater fetal activity (*r* = −0.485, *p* = 0.024).

**Table 4:** Correlation table of bio-measures vs. fetal behavior measures.

Bio-measures		Jerky Movements	Spontaneous Activity/Min	Quiet Bout Length (min)
**MAT 5HT**	r_S_	0.108	−0.456	0.431
**ng/mL**	1-tail p	0.340	0.033	0.042
	*(n)*	(17)	(17)	(17)
**INF 5HT**	r_S_	−0.098	−0.402	0.485
	1-tail p	0.708	0.055	0.024
**ng/mL**	*(n)*	(17)	(17)	(17)
**MAT**	r_S_	0.032	0.086	−0.137
**Plasma SRI**	1-tail p	0.452	0.372	0.300
**ng/mL**	*(n)*	(17)	(17)	(17)
**INF**	r_S_	0.312	−0.176	0.097
**Plasma SRI**	1-tail p	0.129	0.266	0.366
**ng/mL**	*(n)*	(15)	(15)	(15)
**SRI: Est**.	r_S_	0.150	−0.162	0.184
**3rd Trim**.	1-tail p	0.283	0.268	0.240
**Exposure**	*(n)*	(17)	(17)	(17)

r_S_ Spearman’s correlation.

The correlations for infant or maternal plasma SRI levels and the behavioral measures were notably lower. With one exception (infant SRI concentration vs. Jerky movements: *r* = 0.312, *p* = 0.129), all absolute correlations between SRI levels and behavior were less than 0.176 and all *p*-values were greater than 0.266 (Table 4). Similarly, none of the correlations between estimated third trimester SRI dose and fetal behavior measures approached statistical significance.

**Fig. 2:**
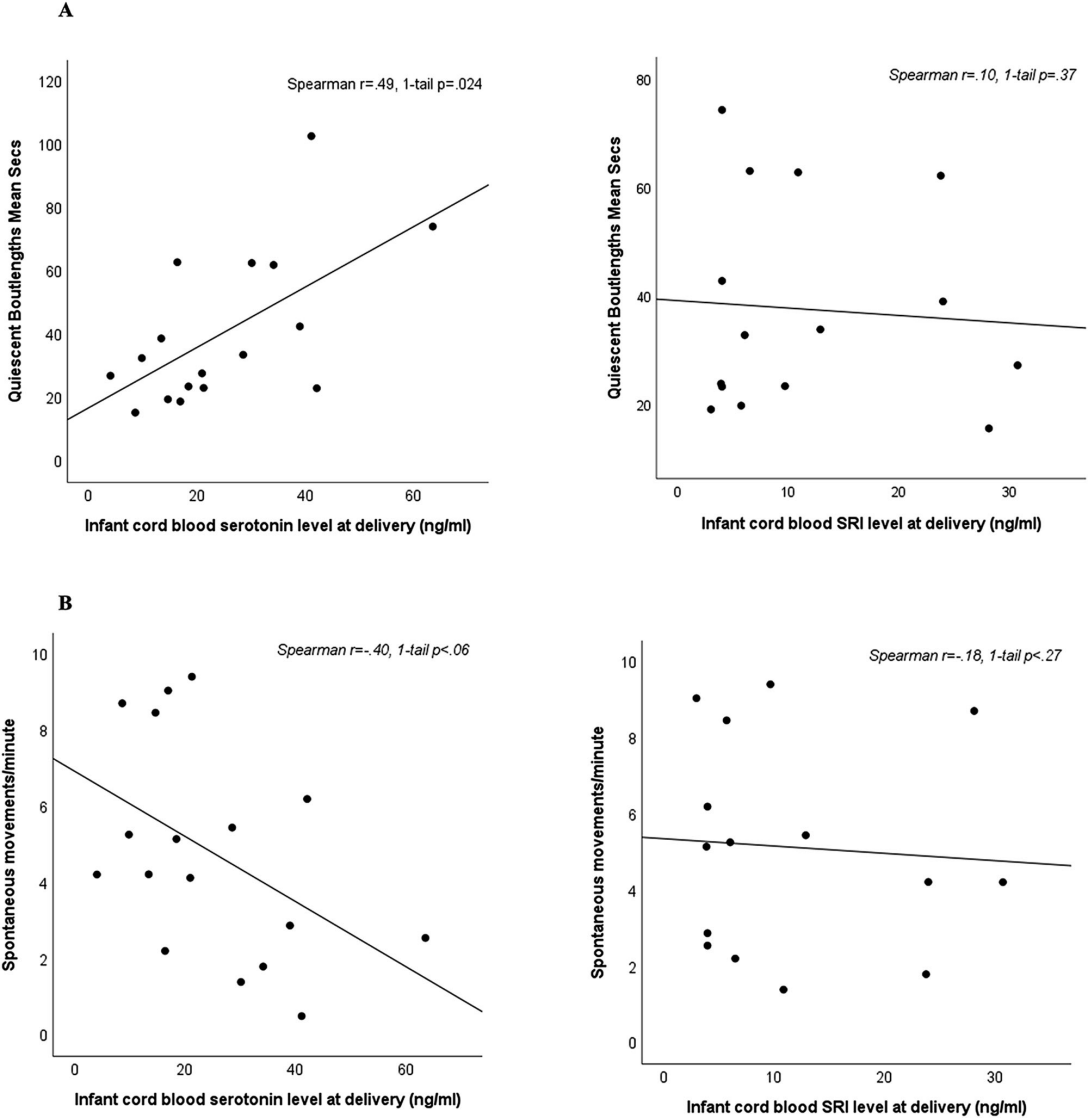
Fetal behavior relationships with infant cord blood serotonin and SRI concentrations. **A** Fetal quiescent boutlengths compared to infant cord blood serotonin at delivery (left panel) and compared to infant cord blood SRI levels (right panel). **B** Fetal spontaneous activity boutlengths compared to infant cord blood serotonin at delivery (left panel) and compared to infant cord blood SRI levels (right panel).

Scatterplots are shown in Fig. 2: 2A is the plot of maternal platelet 5-HT vs. fetal activity compared to the plot of maternal drug level vs. fetal activity. Figure 2B is the plot of maternal platelet 5-HT vs. quiescent bout length compared to the plot of maternal drug level vs. quiescent bout length.

To ensure that the relationships were not changed by the presence of concomitant medications, we also conducted partial correlations using concomitant psychotropic medication status [yes (*n* = 7) vs. no (*n* = 10)] in the 3rd trimester as a control variable. This did not change the overall results of the hypothesis testing but did increase the size of the associations between 5HT and fetal behaviors (Supplemental Figs. S1A and S1B).

## Discussion

### Descriptive statistics

Five of the demographic variables showed significant differences across the three study groups (DEP, DEP + SRI, ND). However, of the five variables, only African American (AA) ancestry would be expected to have an effect on platelet 5-HT levels. Mean platelet 5-HT levels are reported to be 25% higher in AA vs. European American ancestry groups [34]. While the greater proportion of mothers reported AA ancestry in the DEP + SRI group (4 of 17 vs. 1 of 15 in the DEP group and 2 of 28 in the ND group), any effect of AA ancestry would have tended to increase 5-HT levels in the DEP + SRI group and slightly reduce the apparent SRI bioeffect. This is an important consideration, however, sample size in this exploratory study was not high enough to further evaluate this finding.

### Platelet 5-HT group comparisons

As predicted, median maternal and infant platelet 5-HT values in the drug exposed group (DEP + SRI) were significantly lower than in the non-exposed maternal and infant groups (ND, DEP, and combined ND&DEP groups). The 87–88% lower median 5-HT values seen in the DEP + SRI group compared to the combined ND&DEP group were consistent with the 75–90% reductions typically seen in SRI treatment studies [14]. Median 5-HT values were also substantially lower in drug-exposed infants, with values about 72% lower than the non-exposed comparison group. The results are similar to the 75% lower platelet 5-HT levels reported for infants gestationally exposed to SRIs [15]. Furthermore, the data are consistent with the observation that the mean plasma drug level in infants was 62% lower than their mothers.

### Maternal vs. infant 5-HT levels

The median 5-HT values observed for mothers were compared to their infants in each of the DEP, ND, and combined ND&DEP groups. In the non-drug exposed groups, median infant levels were approximately half of maternal levels. This is consistent with previous data reporting lower newborn 5-HT levels compared to older infants and adults, with platelet 5-HT concentrations increasing rapidly after birth and reaching adult levels after one month [11]. In the apparent absence of age-related changes in platelet count or uptake kinetics, the increase with age could be due to increased gut production of 5-HT (thereby increasing platelet exposure to 5-HT and platelet 5-HT levels) as digestive processes mature [35–37]. Finally, maternal and infant 5-HT levels were similar in the DEP + SRI group, which appears to be a consequence of infant 5-HT levels starting lower than maternal levels, while also showing less SRI bioeffect.

### DEP vs. ND 5-HT levels

Maternal and infant comparisons of the median 5-HT values seen for the ND groups vs. the corresponding DEP groups revealed no significant differences (Table 2). Many prior studies of adults have reported either an increase, decrease, or no difference in platelet 5-HT when comparing depression vs. control groups (as recently reviewed [34, 38–40]). The similarity observed here between the ND and DEP groups is consistent with our recent report [34] and further supports the conclusion that platelet 5-HT is not altered in individuals with depression if not receiving an SRI. This result coupled with the prior report [34], indicates that it is not likely that aspects of peripheral 5-HT neurochemistry contributing to platelet 5-HT levels (including gut 5-HT production, 5-HT clearance, and platelet 5-HT uptake [38]) are altered in depression.

### Intercorrelations of drug exposure biomeasures

The correlations observed in the DEP + SRI group for the biomeasures were in the directions expected and nearly all were significant (Table 3). The positive correlations between maternal and infant platelet 5-HT, and between maternal and infant plasma drug concentrations were strikingly high. The invariably inverse relationships observed between the platelet 5-HT measures and the estimated third trimester exposure and plasma drug concentration support the face and construct validities of the platelet 5-HT bioeffect measures. The correlations are congruent with the known inhibitory effects of SRIs on the 5-HT transporter.

Spearman’s correlations between parent and child phenotypes often have been used to estimate trait heritabilities [41]. Maternal-infant correlations in the non-exposed group are not influenced by SRI drug effects and reflect the heritability of the platelet measure. The maternal-infant correlation obtained for the 5-HT ng/mL measure was 0.15 providing an approximate narrow heritability (h^2^ = 2r) estimates of 0.30. Prior studies of platelet 5-HT (ng/mL) heritability have reported narrow heritability estimates of 0.51 [41]. The lower heritability observed here for the ng/mL measure may reflect the relatively small group size, differences between the sampled populations, or the very early infant sampling time-point.

The substantial associations observed between the estimated drug exposure measure and the maternal drug level or the maternal platelet bioeffect measures support the validity of the SDE measure.

#### Biomeasure-fetal behavior correlations

The only biomeasure-behavior correlations that reached or approached statistical significance were observed between maternal or infant platelet 5-HT measures and the frequency of fetal movement (activity per minute) and the quiescent bout-lengths. As expected, lower platelet 5-HT, which reflects greater transporter inhibition due to greater SRI bioeffect, was associated with a higher rate of spontaneous movements and shorter quiescent boutlengths. (see Table 4 and Fig. 2A, B). The phenotype of increased rates of third trimester fetal movement and shorter periods of quiescence associated with greater SRI bioeffect is similar to the restless or fidgety infant behavior associated with the Neonatal Adaptation Syndrome. However, the SRI-associated phenotype might also reflect neurodevelopmental alterations in sleep-related systems which are known to be affected by 5-HT. These findings are present in fetuses when the SRI inhibitory bioeffect is operating, unlike the situation after birth when infants have been suggested to be adapting to reductions in SRI bioeffect. When controlling for concomitant psychotropic medications, the associations between behavior and infant platelet 5HT were higher for fetuses without concomitant exposure, suggesting that other medication exposure may alter these associations and needs to be explored further in future studies.

While the behavioral-platelet 5-HT correlations reached or approached significance, behavioral correlations with the maternal and infant plasma drug levels and estimated third trimester SRI use were consistently and substantially lower (see Table 4). As reviewed in the Introduction, platelet 5-HT provides an index of bioeffect at SERT and was posited to be more highly associated with behavioral effects than plasma drug concentrations or estimates of drug exposure based on dosage. Sertraline and most SRIs are highly protein bound and the advantage of indexing free drug levels is especially important during pregnancy when the concentrations of SRI-binding proteins, including albumin, α1-acid glycoprotein, and lipoprotein, are altered [35–37, 42].

### Potential mechanisms of SRI effects on fetal behavior

Most of the fetal behavioral effects of gestational SRI exposure can be presumed to arise from an increased concentration of brain extracellular 5-HT. This leads to altered serotonergic neurotransmission and neuromodulation, and to potential alterations in 5-HT’s growth factor effects. Changes in levels of 5-HT receptor stimulation and altered downstream growth factor effects may contribute to altered neurodevelopment. The relative importance of altered serotonergic signaling vs. altered neurodevelopment remains to be clarified. However, given the temporal proximity, factors underlying fetal behavior also play a role in neonatal behavior. Evidence for changes in infant behavior through at least one month [13] suggested that neurodevelopmental changes contribute to fetal, neonatal, and infant behavioral alterations. Therefore, while some neonatal behavioral changes might be due to SRI discontinuation phenomena, the relevant processes are operating in the context of the preexisting changes to serotonergic function and/or neurodevelopment.

### Limitations

Rigorous inclusion criteria were used for the study to ensure interpretability of the findings. This resulted in a substantially reduced sample size compared to the larger prospective longitudinal naturalistic cohort study. This did necessarily limit the statistical power of the study in several ways: effects of covariates (such as race/ancestry) could not be definitively tested, and negative findings, (such as fetal sex) may be due to limited sample size. We used one-tailed *p*-values due to the hypothesized direction of effect. In general, the study needs to be considered preliminary and exploratory due to low sample size and power. However, the consistently higher r_S_ values seen for the bioeffect-behavioral correlations compared to the drug level-behavioral correlations provide substantial support for the potential utility of the bioeffect measure in assessing gestational SRI exposure.

## Summary/conclusions

The major findings include: (1) the high degree of correlations among the biomeasures provide substantiation of their validity; (2) the presence of similar and substantial SRI inhibitory bioeffect in mothers and infants; (3) the observation of correlations of both maternal and infant platelet 5-HT with fetal behaviors; and (4) the relatively weaker associations the drug exposure measures and the various fetal and infant behaviors. Taken together, the findings indicate that platelet 5-HT is superior to plasma drug concentrations or other estimates of drug exposure when assessing and interpreting the effects of gestational SRI exposure on fetal and infant behavior.

The observed associations of SRI bioeffect with altered fetal behavior, coupled with reports of the association of SRIs exposure with altered fetal behavior [12, 43, 44] and changes in fetal physiology, indicate that SRI-associated neonatal behavioral alterations are not solely due to drug discontinuation. More generally, SRI-induced increases in brain extracellular 5-HT might affect behavior via growth factor effects on neurodevelopment or by way of receptor mediated effects, or both. Ascertaining the relative importance of these pathways to SRI-induced alterations of fetal, neonatal, and infant behavior requires further research that should benefit from the measurement of maternal and neonatal platelet 5-HT.

## Supplementary information

Figure S1
