# Supplementary figures and images for: Fetal behavior and gestational serotonin reuptake inhibitor exposure: relationships between behavior, drug dosage, plasma drug level, and a measure of drug bioeffect
Source: Neuropsychopharmacology. 2024 Aug 10;49(13):1968–77. doi: 10.1038/s41386-024-01923-1 (PMC11480508; doi:10.1038/s41386-024-01923-1)

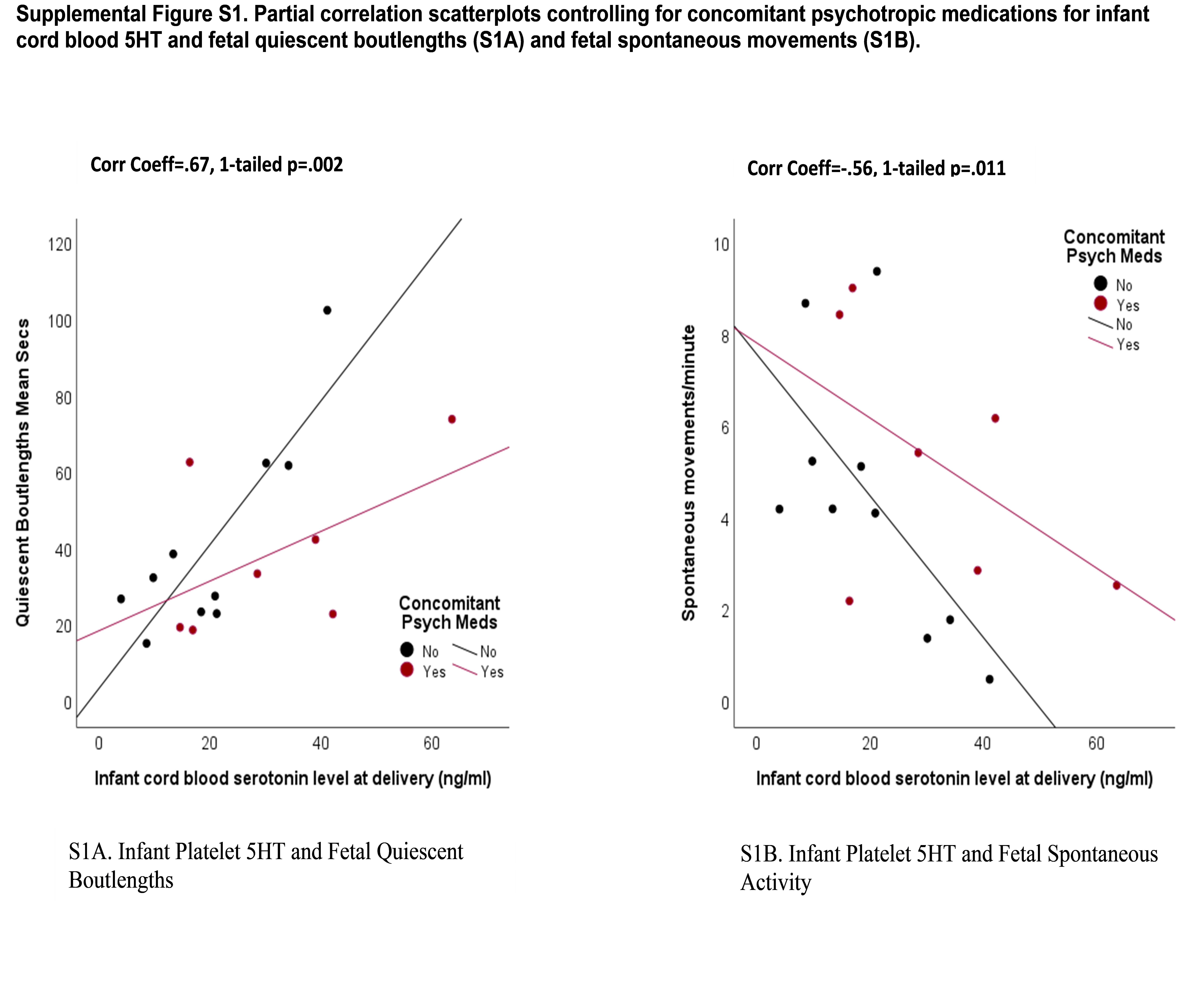

Supplement: Supplementary file 1 — Figure S1 [file 41386_2024_1923_MOESM1_ESM.png]
